# MESENCHYMAL CELLS IN ROTATOR CUFF REPAIR - TECHNIQUE DESCRIPTION AND CASE REPORTS

**DOI:** 10.1590/1413-785220233105e268392

**Published:** 2023-12-18

**Authors:** EDUARDO ANGELI MALAVOLTA, VINICIUS LAMBOGLIA MICELI, JORGE HENRIQUE ASSUNÇÃO, FERNANDO BRANDAO ANDRADE-SILVA, MAURO EMILIO CONFORTO GRACITELLI, NELSON HIDEKAZU TATSUI, LUIZ CÉSAR ESPIRANDELLI, ARNALDO AMADO FERREIRA

**Affiliations:** 1 Universidade de Sao Paulo, Faculdade de Medicina, Hospital das Clinicas HC-FMUSP, São Paulo, SP, Brazil; 2 Hcor, São Paulo, SP, Brazil; 3 Criogenesis Biotecnologia Ltda, São Paulo, SP, Brazil

**Keywords:** Rotator Cuff, Arthroscopy, Mesenchymal Stem Cell Transplantation, Manguito Rotador, Artroscopia, Transplante de Células-Tronco Mesenquimais

## Abstract

**Objective:**

To describe a protocol of obtention of mesenchymal stem cells and to report their use as a biological adjuvant in three patients undergoing arthroscopic rotator cuff repair.

**Methods:**

Case series of patients who underwent arthroscopic repair of isolated full-thickness supraspinatus tear using mesenchymal stem cells obtained from the bone marrow as a biological adjuvant. All patients were operated on at the same institution, by a surgeon with 13 years of experience. The cells were applied at the end of the procedure, at the tendon-bone interface, at an approximate concentration of 2,000,000 mesenchymal cells/mm^3^ and a total volume of 5 ml.

**Results:**

All patients improved with the procedure, with one excellent and two good results. All cases overcame the minimally important clinical difference. All cases reached tendon healing, without partial or complete re-tears. We observed no complications.

**Conclusion:**

Arthroscopic rotator cuff repair with added mesenchymal cells obtained from bone marrow and submitted to a cell expansion process led to good functional results and healing in all cases in the sample, with no complications. ***Level of Evidence IV, Case Series.***

## RESUMO

**Objetivo:**

Descrever o protocolo de obtenção de células mesenquimais e relatar seu uso como adjuvante biológico em três pacientes submetidos ao reparo artroscópico do manguito rotador.

**Métodos:**

Série de casos de pacientes submetidos ao reparo artroscópico de rotura transfixante do músculo supraespinal utilizando como adjuvante biológico células mesenquimais obtidas da medula óssea. Todos ospacientes foram operados na mesma instituição por um cirurgião com 13 anos de experiência. As células foram aplicadas ao final do procedimento, na interface do tendão com o osso, na concentração aproximada de 2 milhões de células mesenquimais/mm^3^ e volume total de 5 ml.

**Resultados:**

Todos os pacientes melhoraram após o procedimento, havendo um resultado excelente e dois bons. Todos superaram a diferença clínica minimamente importante. Em todos os casos ocorreu cicatrização tendínea, sem a presença de rerroturas parciais ou completas. Não observamos complicações.

**Conclusão:**

O reparo do manguito rotador artroscópico com adição de células mesenquimais obtidas da medula óssea e submetidas a processo de expansão celular levou a bons resultados funcionais e cicatrização, sem complicações, em todos os casos da amostra. **Nível de Evidência IV, Série de Casos.**

## INTRODUCTION

Rotator cuff tear is present in 20% of the population,^1^ and problems related to these tendons represent 64% of consultations with a shoulder and elbow surgeon. ^(^ ^2^ The increasing number of surgical repairs of these lesions^3^ is costly to the health system. ^(^ ^4^ Despite several advances in the technique and in the development of fixation methods, the rate of re-tear after the procedure remains high. ^(^ ^5^

The main cause of failure after rotator cuff repair concerns tissue deficiency and the healing process between the tendon and bone. ^(^ ^6^ ^),(^ ^7^ After the intervention, the rotator cuff does not restore its original histological characteristics and its fixation occurs by scar tissue^8^ with lower biomechanical resistance. ^(^ ^9^ Trying to improve structural outcomes after rotator cuff repair, biological adjuvants are studied, such as platelet-rich plasma, ^(^ ^10^ bone marrow stimulation, ^(^ ^11^ grafts, ^(^ ^12^ and mesenchymal cells, ^(^ ^13^ ^), (^ ^14^ ^), (^ ^15^ ^), (^ ^16^ ^), (^ ^17^ ^), (^ ^18^ ^), (^ ^19^ still without a consensus in the literature on their effectiveness.

This study aims to describe the protocol for obtaining mesenchymal cells and to report their use as a biological adjuvant in 3 patients undergoing arthroscopic rotator cuff repair, in addition to evaluating their safety and possible complications.

## METHODS

We treated a series of cases of patients submitted to arthroscopic repair of full-thickness tear of the supraspinatus, using mesenchymal cells obtained from the bone marrow as a biological adjuvant. All patients were operated in the same institution, by a surgeon with 13 years of experience, in 2019. The research protocol was approved with number 77866417.8.0000.0068 and the participants filled out the informed consent form.

Inclusion criteria were: full-thickness tear of the supraspinatus tendon, with retraction of less than 30 mm; pain and/or decreased shoulder strength for at least 3 months, with no improvement with nonsurgical treatment; fatty degeneration of the rotator cuff muscles of grade 1 or 2 according to the classification of Fuchs et al., ^(^ ^20^ absence of tear of the subscapularis or infraspinatus, and skeletal maturity. We did not include pregnant patients nor those with shoulder arthrosis, previous shoulder surgeries or fractures, psychiatric diseases, fibromyalgia, painful pathologies of the cervical spine, rheumatic diseases, chronic use of corticosteroids, active or recent infection, coagulopathies, vascular or neurological lesions, thrombocytopenia, coagulopathies, chronic use of anticoagulants, or comorbidities not clinically compensated.

### Intervention

The patients underwent to general anesthesia associated with interscalene block of the brachial plexus and positioned in beach chair position. Asepsis was performed with 4% chlorhexidine solution, followed by antisepsis with alcoholic solution of the same product. Antibiotic prophylaxis was performed with cefazolin 2 g every 8 hours for a period of 24 hours. The conventional, posterior, anterior, and lateral portals were performed. For placing the anchors, accessory portals were made, in a position that allowed their introduction with an appropriate angle of attack. The procedure was performed without cannulas, except for the moment of the knots.

**Figure 1:**
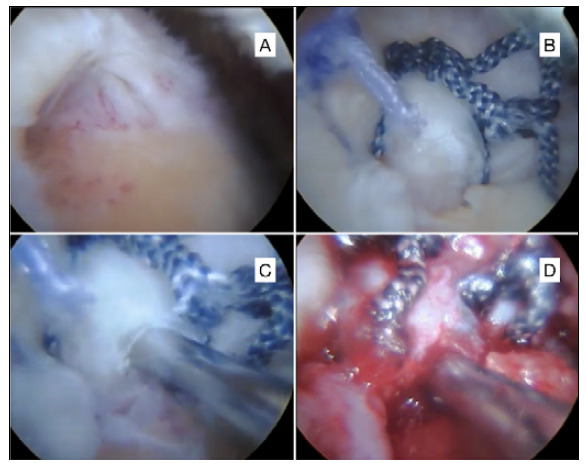
A: Rotator cuff tear before repair; B: Rotator cuff repair; C: Catheter positioning at the tendon-bone interface; D: Joint after aspiration of excess saline.

Bursectomy was performed in all cases. The tendon of the long head of the biceps was approached when it presented instability or partial injury greater than 50%, with tenotomy or tenodesis with anchors in the bicipital groove. The greater tuberosity was debrided until it was free of tendon stumps and bursal tissue, presenting a good site for tendon reinsertion (Figure 1A). No patient underwent distal resection of the clavicle. The rotator cuff was repaired next to the greater tubercle using anchors of 5 mm in diameter. The rotator cuff was repaired using double loaded 5mm anchors, in single-row and with simple stitches (Figure 1B). After suturing the tendon, a Jelco^©^ 14 catheter was positioned at the tendon-bone interface (Figure 1C), then the excess saline solution was aspirated from the subacromial space (Figure 1D) and the arthroscopic portals were sutured with simple stitches, using nylon threads number 4-0.

### Mesenchymal cells

#### Mesenchymal cells collection

The cells were collected by puncture of the sternum medullary region, under local anesthesia and sterile conditions, obtaining around 30 ml of bone marrow. This amount was divided into 3 to 4 syringes containing 1,000 units of heparin each. The material was immediately sent to the cell therapy laboratory to begin the culture.

#### Culture medium preparation

Autologous Platelet-Rich Plasma (PRP) was used as culture medium, replacing the generally used Fetal Bovine Serum. The PRP was obtained by the apheresis method, using the Haemonetics MCS plus cell separator and the disposable kit for collection of single-donor platelet concentrate 994CF-E (Haemonetics Corp, USA). Initially, the apheresis material was mounted on the cell separator and the apparatus circuit was filled with sodium citrate (anticoagulant solution). The proportion of sodium citrate used was 1 ml for every 9 ml of blood. After preparation, information on the number of cycles to be performed, sex, weight, and height of the donor were provided to the device program to calculate the volume of blood and the number of platelets to be collected. After the peripheral venipuncture, 1 ml of the volunteer’s blood was withdrawn for the complete blood count. After receiving the control of hematimetric levels, 400 to 450 ml of blood were drained into the separation device, under continuous centrifugation, at 4,500 rpm for approximately 10 minutes. In the device, the blood was separated into several phases by centrifugation. An optical analyzer located at the apex of the device detected, by refraction, first the platelet-poor plasma (PPP) layer then, upon detecting the platelet layer, the device commanded the collection of this desired component for our therapeutic procedure to a specific bag for blood component, sterile and under a biologically closed system. Around 2 to 4 cycles were performed. At the end, the rest of the blood components returned to the patient through the same venous access. Blood count was performed on the patient and the product collected after the procedure. To the platelet product, 400 micrograms of 10% calcium chloride was added for every 10 ml of platelet concentrate. After 60 minutes, with the formation of the clot and its physiological retraction, the remaining fluid and yellowish color supernatant was extracted. After filtration, with a 0.22 micra barrier, 1 ml of the product was collected for each culture bottle of the Bact-Alert system. The remainder was preserved at −80° Celsius.

#### Cell culture

The bone marrow aspirate was handled in a class II biological safety booth, within a class 10,000 laboratory environment and in a clinic approved for the Brazilian Health Regulatory Agency (ANVISA)/group II cell therapy category. The aspirate was mixed with 4 volumetric parts of DPBS (Dulbecco Phosphate-buffered saline; GIBCO, Grand Island, NY) in 3 or 4 Falcon tubes of 50 ml. After centrifugation at 900 g for 10 minutes at 20°C, surface layers were transferred to another container with 25 ml of Percoll at a density of 1,073 g/ml and cell concentration not exceeding 2 × 10^7^ (Sigma, St. Louis, MO). These samples were submitted to 900 g for 10 minutes at 20°C. The mononuclear cells were resuspended in DPBS and centrifuged at 460 g for 10 minutes at 20°C. The cells were again resuspended at a concentration of 1 × 10^6^ nucleated cells per milliliter of DMEM low Glucose (Dulbecco modified Eagle medium, low glucose, Gibco), 10% serum from autologous platelet-rich plasma and 1% non-essential amino acid (NEAA), L-Glutamine proportion of 1% and antibiotic with antimycotic proportion of 1%. Around 30 ml of suspension were plated per bottle of 175 cm^2^ or 75 cm^2^ (Falcon, Franklin Lakes, NJ). The culture bottles were grown in incubators with a controlled environment at 5% CO_2_. Culture media were changed every 24 to 48 hours. When the culture reached around 90% confluence, the adherent cells were detached with 0.05% trypsin (Gibco) and the passage was made respecting the concentration of 1 × 10^6^ per bottle. After 2 passages, they were processed for surgical use.

#### Flow cytometry

To demonstrate the immunological characteristics and the homogeneous population obtained after the expansion culture of mesenchymal stem cells, a small aliquot containing at least 4 × 10^4^ cells was evaluated for the expression of surface markers, namely: CD 105 FITC clone: 43A3 (BD Pharmigen, San Diego, CA), CD90 PE clone: 5E10 (BD Pharmigen, San Diego, CA), CD34 PE clone: 581 (BD Pharmigen, San Diego, CA), CD45 FITC clone: HI30 (BD Pharmigen, San Diego, CA). The analysis was performed in FACScan (Beckton Dickinson), and the data were analyzed with the CellQuest program (Beckton Dickinson). The immunophenotyping assay for mesenchymal stem cell culture expressed negativity for CD45 and CD34 and positivity for CD90 and CD105. The results were expressed by histogram. The markings with positive expressions had to reach > 90%, otherwise the sample was discarded.

#### Cell count

Around 0.5 ml of the final product was submitted to cell counting by the manual method using the Neubauer chamber and optical microscopy.

#### Contamination by bacteriological agents

Before the surgical use of mesenchymal stem cells, an aliquot of 2 ml was evaluated for possible bacterial or fungal contamination. The Bact-Alert automated culture system (bioMérieux, Durham, NC) was used. Samples that showed a possible contaminant were discarded.

#### Cryopreservation of an aliquot

At least one representative cell sample of the material was cryopreserved with 2 ml of the following cryoprotective solution: DMEM F12 or DMEM low glucose 70%, Hyclone 20% and DMSO 10%. With a pipette, the cryoprotective solution was aspirated and added to the container containing the mesenchymal stem cells. Programmable freezing was performed with the Cryomed 1010 system. After the freezing was finished, the container was placed in nitrogen vapor at a temperature below −160°C.

#### Surgical use

**Figure 2:**
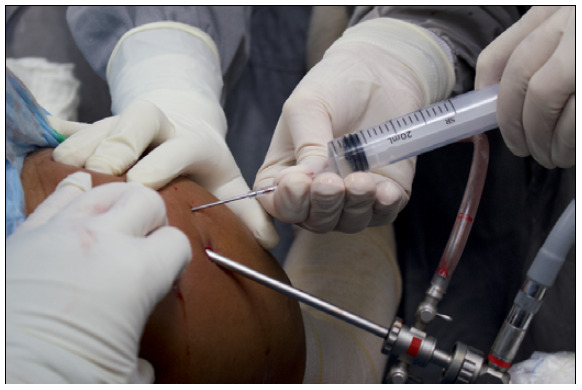
Application of mesenchymal cells, through a catheter positioned at the tendon-bone interface, while the auxiliary occludes the arthroscopic portals.

The cells were transported in a sterile and apyrogenic container. The transport temperature was maintained between 20 and 25°C. The means of transport was the PRP obtained from the patient. After closing the saline flow in arthroscopy and emptying the excess saline from the subacromial space, the material containing mesenchymal cells and PRP, totaling 5 ml and with an approximate concentration of 2,000,000 mesenchymal cells/ml was applied to the tendon-bone interface, through a previously positioned Jelco^©^ catheter following the same protocol of previous studies conducted by our group using platelet-rich plasma. ^(^ ^21^ ^),(^ ^22^ During this process, an assistant kept the already sutured portals compressed, to avoid extra leakage of the material (Figure 2).

#### Postoperative care and rehabilitation

During hospitalization, patients were maintained on intravenous medications, those being an analgesic (Dipyrone 2 g every 6 hours), a non-hormonal anti-inflammatory drug (Ketoprofen 100 mg every 12 hours), and an opioid analgesic (Tramadol 100 mg every 8 hours). After discharge, the medication was administered orally and consisted of Dipyrone 2 g every 6 hours for 10 days and Tramadol 50 mg every 6 hours for 5 days. After this period, the need for medication was individualized. The patients were discharged the day after surgery. The dressing was changed on the 1^st^ day and kept closed until the return, 7 days after surgery.

Velpeau-type immobilization was used for 6 weeks, and no movement was performed with the shoulder in the first 3 weeks. Movements with the elbow, wrist, and fingers were oriented. After the end of the third week, passive exercises started. The assisted active and free active exercises started after the sixth week, alongside stopping the use of the sling. Muscle reinforcement, with active resistance exercises, was performed only after the significant gain of movement, in the twelfth week.

#### Outcomes

Patients were clinically evaluated using the University of California at Los Angeles (UCLA) scale, ^(^ ^23^ 1 week before surgery and at 24 months. The patients underwent magnetic resonance imaging (MRI) before the procedure and 6 months after. The tests were performed on a GE HDxt® 1.5 Tesla device (General Electric Corp, USA). The postoperative aspect of the tendon was described according to the classification of Sugaya et al., ^(^ ^24^ which stratifies the aspect of the tendon after repair into 5 levels: type I (sufficient thickness with low signal in all sections); type II (sufficient thickness with high focal signal); type III (insufficient thickness without discontinuity); type IV (small size tear); and type V (medium or large size tear).

## RESULTS

We performed three rotator cuff repairs adding mesenchymal cells. Table 1 shows the general characteristics of the sample.

**Table 1:** General sample characteristic.

	Sex	Age	Comorbidity	Biceps procedure	Anchors	Retraction (mm)	Extension (mm)
Patient 1	F	58	Diabetes	None	2	20	16
Patient 2	M	59	Hypertension	Tenotomy	2	12	11
Patient 3	M	61	None	Tenodesis	1	10	8

**Figure 3:**
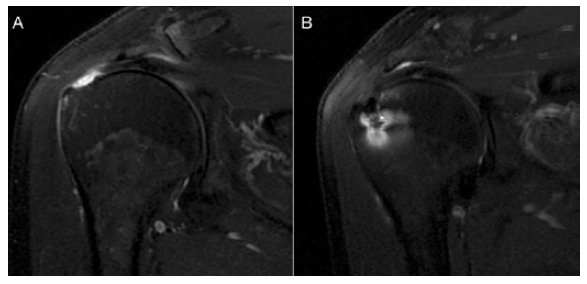
MRI. A: Preoperative oblique coronal image demonstrating full-thickness tear of the supraspinatus; B: Complete repair, with Sugaya type I classification (sufficient thickness with low signal in all cuts).

All patients improved with the procedure. According to Ellman’s classification, we had one excellent result and two good ones, all of which overcame the minimally important clinical difference. ^(^ ^25^ In all cases, tendon healing occurred without partial or complete tears. Table 2 and Figure 3 show the data. We observed no complications.

**Table 2:** Postoperative results.

	UCLA pre-op	UCLA 24m	Sugaya
Patient 1	16	31	Type II
Patient 2	11	30	Type I
Patient 3	27	35	Type I

## DISCUSSION

In this study, we describe the clinical and structural results of patients submitted to arthroscopic repair of the rotator cuff with added mesenchymal cells. All patients had significant functional improvement by the UCLA scale, surpassing the minimally important clinical difference. ^(^ ^25^ All cases also showed tendon healing.

Few comparative studies to date have evaluated the effect of mesenchymal cells on rotator cuff repair, including three randomized^13^ ^),(^ ^16^ ^),(^ ^19^ and two cohorts. ^(^ ^15^ ^),(^ ^18^ Šmíd et al. ^(^ ^19^ observed significantly greater clinical and structural improvement in the group that received mesenchymal cells during open repair, in a randomized study involving 50 patients. Randelli et al. ^(^ ^13^ observed clinical superiority only at 6 months, with no difference in the other follow-up times or in the image analysis, in a randomized study involving 44 patients undergoing arthroscopic repair. In turn, Lamas et al. ^(^ ^16^ observed no differences between the groups, when analyzing a sample of only 13 patients submitted to open repair.

Hernigou et al., ^(^ ^18^ in a paired cohort study involving 90 patients, observed a significant reduction in the number of tears in the group submitted to mesenchymal cell application, without evaluating functional outcomes. Kim et al., ^(^ ^15^ on the other hand, despite not noticing functional differences between the groups, also reported better structural results with the use of mesenchymal cells.

Thus, although the literature shows no consensus and no meta-analyses compiling the data, we can observe that most studies demonstrate effectiveness of the use of mesenchymal cells as biological adjuvants to rotator cuff repair.

We observed no complications in our study. These data agree with those reported by Randelli et al., ^(^ ^13^ who did not describe any complications in the 22 patients submitted to the application of mesenchymal cells. Lamas et al., ^(^ ^16^ however, discontinued their study early due to the high number of complications, 23%, against 8% in the control group. They describe the formation of subacromial inflammatory tissue, consisting of intense synovitis and granulomatous tissue.

Mesenchymal cells can be obtained from bone marrow^16^ ^),(^ ^18^ ^),(^ ^19^ or from adipose tissue, ^(^ ^13^ ^),(^ ^15^ and our protocol used the first option. Note that we obtained the cells by puncture of the sternum, unlike Lamas et al. ^(^ ^16^ and Hernigou et al., ^(^ ^18^ who punctured the iliac crest, and Šmíd et al., ^(^ ^19^ who used the humeral head.

Our protocol performed cell culture and expansion to apply a known and high concentration of cells at the time of surgery (10 × 10^6^ cells). This procedure was performed only by Lamas et al., ^(^ ^16^ where about 20 × 10^6^ cells were used. Our culture time, however, was longer (4 vs. 2 weeks) and we cryopreserved an aliquot, allowing future expansion and application. The other studies analyzed did not perform cell expansion, and applied cells obtained at the time of surgery. ^(^ ^13^ ^),(^ ^15^ ^),(^ ^18^ ^),(^ ^19^ We believe that high concentrations of mesenchymal cells, made possible by cell culture, as well as cryopreservation, are highly beneficial in procedures involving cell therapy.

Our study has some limitations. In particular, this being a case series with few patients. In addition, the use of metallic anchors impairs the visualization of tendon healing, and we did not perform a new arthroscopy to collect anatomopathological material that confirms tendon regeneration. However, we describe a protocol with cell culture and expansion, which allows to apply a large number of cells in patients with a clinical follow-up of 24 months and structural evaluation by magnetic resonance imaging. Further randomized studies and meta-analyses are needed to determine the effectiveness of the use of mesenchymal cells in rotator cuff repair.

## CONCLUSION

Arthroscopic rotator cuff repair with added mesenchymal cells obtained from bone marrow and submitted to a cell expansion process led to good functional results and healing in all cases in the sample, with no complications.
